# Improving Nocturnal Hypoxemic Burden with Transvenous Phrenic Nerve Stimulation for the Treatment of Central Sleep Apnea

**DOI:** 10.1007/s12265-020-10061-0

**Published:** 2020-08-12

**Authors:** Olaf Oldenburg, Maria Rosa Costanzo, Robin Germany, Scott McKane, Timothy E. Meyer, Henrik Fox

**Affiliations:** 1 Clinic for Thoracic and Cardiovascular Surgery, Herz- und Diabeteszentrum NRW, Ruhr-Universität Bochum, Bad Oeynhausen, Germany; 2 Ludgerus-Kliniken Münster, Clemenshospital, Münster, Germany; 3 Heart Failure Department, Herz- und Diabeteszentrum NRW, Ruhr-Universität Bochum, Georgstr. 11, D-32545 Bad Oeynhausen, Germany; 4 Advocate Heart Institute, Naperville, IL USA; 5 Respicardia, Inc, Minnetonka, MN USA

**Keywords:** Central sleep apnea, Phrenic nerve stimulation, Hypoxemic burden

## Abstract

Nocturnal hypoxemic burden is established as a robust prognostic metric of sleep-disordered breathing (SDB) to predict mortality and treating hypoxemic burden may improve prognosis. The aim of this study was to evaluate improvements in nocturnal hypoxemic burden using transvenous phrenic nerve stimulation (TPNS) to treat patients with central sleep apnea (CSA). The remedē System Pivotal Trial population was examined for nocturnal hypoxemic burden. The minutes of sleep with oxygen saturation < 90% significantly improved in Treatment compared with control (*p* < .001), with the median improving from 33 min at baseline to 14 min at 6 months. Statistically significant improvements were also observed for average oxygen saturation and lowest oxygen saturation. Hypoxemic burden has been demonstrated to be more predictive for mortality than apnea–hypopnea index (AHI) and should be considered a key metric for therapies used to treat CSA. Transvenous phrenic nerve stimulation is capable of delivering meaningful improvements in nocturnal hypoxemic burden. There is increasing interest in endpoints other than apnea–hypopnea index in sleep-disordered breathing. Nocturnal hypoxemia burden may be more predictive for mortality than apnea–hypopnea index in patients with poor cardiac function. Transvenous phrenic nerve stimulation is capable of improving nocturnal hypoxemic burden.

**Figure:**
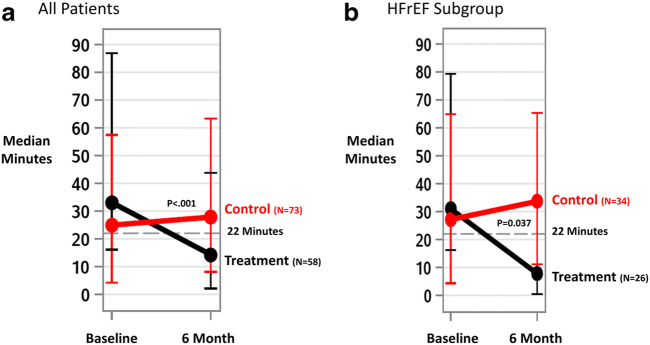
Graphical Abstract

Graphical Abstract

## Introduction

The apnea–hypopnea index (AHI), defined as the number of apnea and hypopnea events per hour of sleep, has been the most commonly used measure to describe the burden of sleep apnea [1]. The AHI was initially developed for use in measuring severity of obstructive sleep apnea (OSA) and then was adopted for central sleep apnea (CSA), even though the mechanisms and deleterious impacts of the disorders are very different [2]. CSA does not always result in significant oxygen desaturation as typically seen in OSA, and the AHI does not adequately describe desaturation by its current definition [3]. Despite the AHI being easy to measure by a sleep laboratory and easy to understand by clinicians for the diagnosis of sleep apnea, questions persist about whether AHI describes the complete picture of the severity of both central and obstructive sleep apnea [3]. Moreover, apneas do not require desaturations to count towards AHI and hypopneas are not always related to desaturation. Thus, the AHI could be very high with little to no oxygen saturation below 90% [3]. Reducing CSA severity leads to improvements in AHI that should translate to improved oxygenation during sleep [4, 5]. A therapy may reduce the impact of each event if the depth of the desaturation is lessened or event duration is shortened, even if some apnea and hypopnea events are not eliminated [6]. A recent study by Oldenburg et al. identified nocturnal hypoxemic burden, defined as the time a patient spends with an oxygen saturation below 90% (T90), as the most robust predictor of survival in patients with sleep apnea and stable heart failure with reduced ejection fraction (HFrEF) [3]. In contrast, the same study demonstrated that AHI alone was a weak predictor of mortality [3]. Thus, the time of oxygen saturation below 90% should be examined for any therapy treating CSA [7]. Device treatments for CSA include mask-based therapies, such as continuous airway pressure [8] and adaptive servo ventilation [7], and a transvenous phrenic nerve stimulation (TPNS) device [9, 10]. However, investigations on treatments for CSA to reduce hypoxemic burden are limited. The **rem**edē System Pivotal Trial investigated patients with moderate to severe CSA and the results demonstrated 60% of patients using transvenous phrenic nerve stimulation experienced a 50% or greater reduction in AHI at 6 months [11]. The effects of TPNS on hypoxemic burden might be even more clinically and prognostically important than AHI reduction but has not been reported in detail yet. This analysis evaluates changes in nocturnal hypoxemic burden with 6 months of TPNS (delivered by the **rem**edē System) versus control (optimal medical management and implanted but inactive **rem**edē system).

## Methods

The **rem**edē System Pivotal Trial was a prospective, multicenter, randomized, open-label controlled trial in patients with predominant CSA of different etiologies to assess transvenous unilateral phrenic nerve stimulation versus no stimulation [12]. The primary results [11], 12-month results [13], long-term results [9], and results in the subgroup of patients with heart failure [14] have been reported. Patients underwent an in-laboratory, attended polysomnogram at baseline and 6 months, both of which were scored by a central and blinded sleep core laboratory (Registered Sleepers, Leicester, NC, USA). The primary and secondary effectiveness endpoints were based on changes in AHI and other sleep indices, as well as quality of life. Data extracted/analyzed from the polysomnograms also included assessment of nocturnal hypoxemic burden, including both minutes of sleep with oxygen saturation < 90% (T90) and percentage of sleep with oxygen saturation < 90%. Other parameters related to oxygenation available from the polysomnogram included the average saturation level, lowest oxygen saturation, and oxygen desaturation index (4%).

The change from baseline to 6 months in sleep metrics and oxygen saturation parameters were compared between groups using the Mann–Whitney test in the per protocol population. Results are presented as median and interquartile range. The analyses were repeated in the subgroup of patients with heart failure and ejection fraction ≤ 45% (HFrEF). A nominal 2-sided *p* < 0.05 will be considered statistically significant in this exploratory analysis. SAS version 9.4 (Cary, NC, USA) was used for all analyses.

## Results

**Fig. 1:**
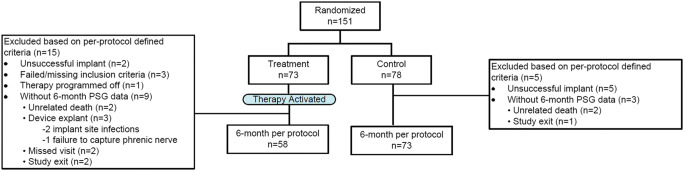
CONSORT Diagram, Composition of the per protocol population with sleep study results through the 6-month visit, PSG = polysomnogram

The Pivotal Trial enrolled 151 patients with predominantly CSA, including 131 (58 treatment and 73 control) who qualified for the per protocol analysis. The primary reason for exclusion from per protocol was no endpoint data at 6 months (12 subjects). Reasons for patient exclusion from the per protocol population and 6-month analysis are displayed in the CONSORT diagram (Fig. 1). Patients in the per protocol population were primarily male 91% (119/131), 61% (80/131) had HF, median left ventricular ejection fraction of 44.0 [29.0, 49.0], and had severe CSA with median AHI of 43.9 [32.3, 60.2] events/h (Table 1).

**Table 1:** Baseline clinical characteristics by randomized groups in the per protocol population

Baseline characteristic	Treatment (*n* = 58)	Control (*n* = 73)
Age (years)	63 ± 12	64 ± 14
Men	51 (88%)	68 (93%)
White	57 (98%)	69 (95%)
Body mass index (kg/m^2^)	30 ± 5	31 ± 7
Neck circumference (cm)	42 ± 5	43 ± 5
Heart rate (beats per minute)	76 ± 13	73 ± 14
Systolic blood pressure (mmHg)	124 ± 18	124 ± 18
Diastolic blood pressure (mmHg)	74 ± 11	75 ± 12
Respiration rate (breaths per minute)	18 ± 3	17 ± 3
Apnea hypopnea index (events/h)	50 ± 19	44 ± 17
Central apnea index (events/h)	32 ± 19	26 ± 16
Obstructive apnea index (events/h)	2 ± 2	2 ± 3
Mixed apnea index (events/h)	3 ± 4	2 ± 3
Hypopnea index (events/h)	13 ± 11	13 ± 12
Percent of Sleep with Oxygen Saturation < 90% (%)	17 ± 18	11 ± 12
Oxygen desaturation ≥ 4% index (events/h)	44 ± 22	37 ± 18
Epworth Sleepiness Scale (points)	11 ± 5	9 ± 6
Atrial fibrillation	22 (38%)	29 (40%)
Left ventricular ejection fraction ≤ 45%	32/57 (56%)	42/70 (60%)
Heart failure	35 (60%)	45 (62%)
New York Heart Association class		
I	5 (9%)	12 (16%)
II	14 (24%)	20 (27%)
III	16 (28%)	13 (18%)
IV	0%	0%
Coronary artery disease	33 (57%)	42 (58%)
Hypertension	42 (72%)	55 (75%)
Diabetes mellitus	20 (34%)	17 (23%)
Prior stroke	4 (7%)	5 (7%)
Renal impairment	11 (19%)	20 (27%)
Concomitant cardiac devices	24 (41%)	30 (41%)
Implantable cardioverter defibrillator	14 (24%)	13 (18%)
Cardiac resynchronization therapy defibrillator	8 (14%)	9 (12%)
Non-cardiac resynchronization therapy pacemaker	2 (3%)	8 (11%)
Angiotensin converting enzyme inhibitor	28 (48%)	35 (48%)
Angiotensin receptor blocker	9 (16%)	13 (18%)
Aldosterone-blocking agent	25 (43%)	17 (23%)
Beta-blocker	36 (62%)	47 (64%)
Loop diuretic	26 (45%)	26 (36%)
Thiazide diuretic	15 (26%)	16 (22%)
Thiazide-like diuretic	5 (9%)	2 (3%)
Antiarrhythmic	4 (7%)	7 (10%)
Digoxin	10 (17%)	12 (16%)

Continuous variables reported as mean ± standard deviation and categorical display *n* (percent)

**Fig. 2:**
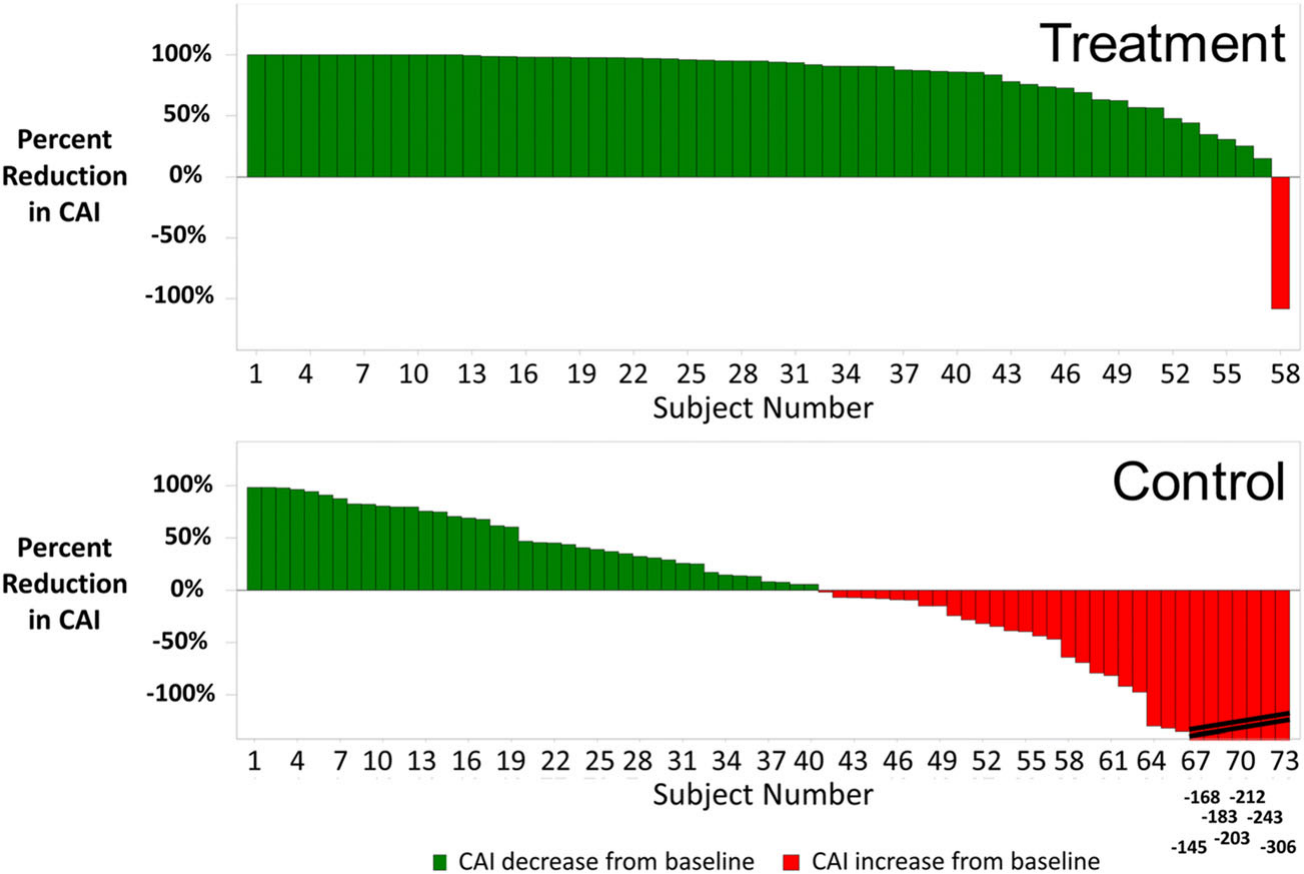
Central Apnea Index Percentage Reduction from baseline to 6 Months for each subject, each vertical bar represents the percentage reduction (improvement) in the central apnea index from baseline to 6 months for a subject. The green bars (> 0%) represent reduction and red (< 0%) represent increase. Actual percentage increase for control subject results beyond the scale are noted at the bottom of the bars, CAI = central apnea index

As previously reported [11], the AHI improved by a median of 23 [− 38, − 10] events per hour in the treatment group and worsened by 1 [− 7, 15] event per hour in control (*p* < .001). Central apnea events, which were the most common event type, were nearly eliminated. Median central apnea index (CAI) was 30 [16, 43] events per hour at baseline in the treatment group and decreased to 1 [0, 7] event per hour at 6 months; the Control group CAI was 21 [14, 35] events per hour at baseline and 22 [10, 35] at 6 months (between group difference for change from baseline *p* < .001) (Table 2). Figure 2 displays the percentage reduction in CAI from baseline for each patient. The oxygen desaturation index (4%) decreased from a median of 41 [30, 56] events per hour to 19 [8, 37] in the treatment group and increased from 33 [25, 50] events per hour to 39 [26, 57] in control (between groups *p* < .001). Marked or moderate improvement in the patient global assessment quality of life instrument was noted by 60% (35/58) treatment versus 6% (4/72) control subjects (*p* < .001) and daytime sleepiness decreased 2.5 [− 7, − 1] points in treatment compared with increasing 1.0 [− 2, 2] points in control based on the Epworth Sleepiness Scale (*p* < .001).

**Table 2:** Median results for sleep parameters from overnight polysomnogram results by visit in the per protocol population

	Treatment (*N*=58)			Control (*N*=73)			Between group *P*-value
Variable	Baseline	6 Months	Change	Baseline	6 Months	Change	
AHI (events/hour)	49 35, 60	21 11, 35	−23 −38, −10	40 31, 54	42 32, 60	1 −7, 15	<.001
CAI (events/hour)	30 16, 43	1 0, 7	−20 −40, −13	21 14, 35	22 10, 35	−3 −11, 8	<.001
ODI4% (events/hour)	41 30, 56	19 8, 37	−19 −33, −3	33 25, 50	39 26, 57	3 −6, 14	<.001
Minutes of Sleep with O2	33	14	−16	25	28	1	<.001
Saturation<90% (minutes)	16, 87	2, 44	−44, 0	4, 58	8, 63	−14, 16	
Percent of Sleep with O2	9	5	−4	9	8	0	0.003
Saturation<90% (%)	5, 27	1, 20	−10, 1	2, 17	2, 19	−5, 5	
Average O2 Saturation	92.6	93.3	0.7	93.0	92.7	0.1	0.006
during sleep (%)	91.3, 94.2	91.9, 94.7	−0.4, 1.7	91.9, 94.4	91.8, 93.9	−0.8, 0.8	
Lowest O2 Saturation	81.0	81.5	2.0	82.0	81.0	−1.0	0.011
during sleep (%)	76.0, 84.0	77.0, 87.0	−3.0, 5.0	77.0, 85.0	77.0, 84.0	−4.0, 3.0	

Reported as median and interquartile range

*P*-value from Mann-Whitney test for difference in change from baseline between groups (2-sided)

*AHI* apnea-hypopnea index; *CAI* central apnea index; *ODI4*% oxygen desaturation ≥4% index; *O2* oxygen

**Fig. 3:**
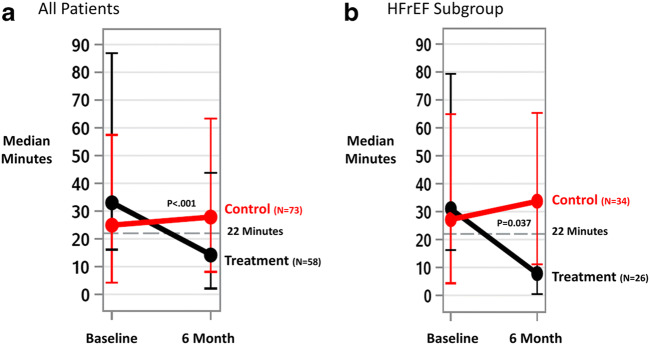
Median (interquartile range) of minutes with oxygen saturation < 90%, median (and interquartile range) minutes with oxygen saturation < 90% at baseline and 6 months for the full per protocol population (**A**) and the subgroup with heart failure and ejection fraction ≤ 45% (**B**). *P* value from Mann–Whitney test for difference in change from baseline between groups (2-sided)

**Fig. 4:**
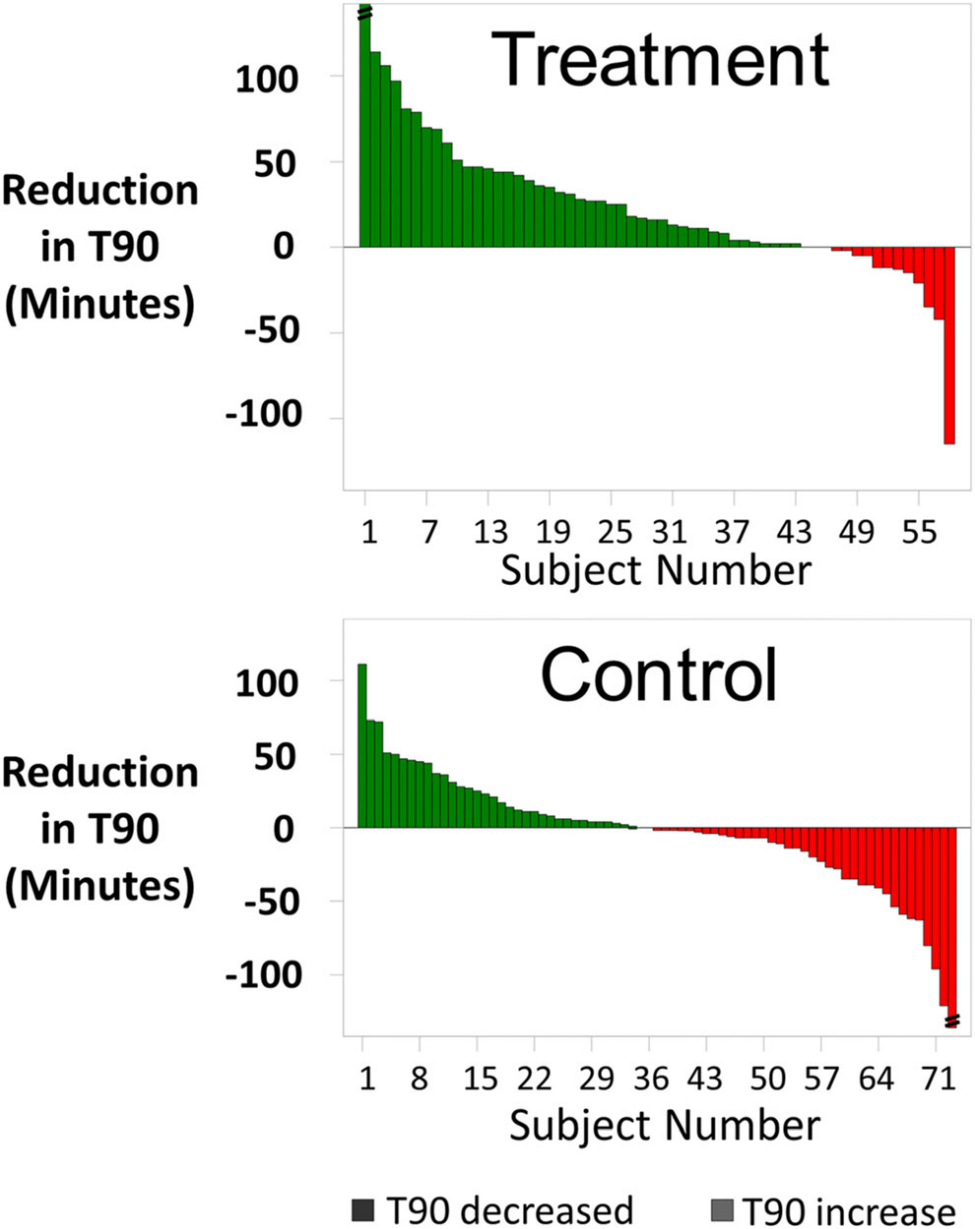
Oxygen saturation < 90% improvement from baseline to 6 months for each subject. Each vertical bar represents the reduction (improvement) in minutes of sleep with oxygen saturation < 90% from baseline to 6 months for a subject. The green bars (> 0%) represent reduction and red (< 0%) represent increase. Actual percentage increase for control subject results beyond the scale are noted at the bottom of the bars, Min = minutes; T90 = minutes of sleep with oxygen saturation < 90%

Nocturnal hypoxemic burden, as measured by minutes of sleep with oxygen saturation < 90%, improved significantly more in the treatment group than in the control group. The median minute with oxygen saturation < 90% during the baseline polysomnogram was 33 [16, 87] min for the treatment group and 25 [4, 58] min for control (Fig. 3A; Table 2). At the 6-month visit, the median in the treatment group was 14 min [2, 44] versus 28 [8, 63] min in control. The median changes from baseline were − 16 [− 44, 0] min for treatment and + 1 [− 14, 16] for control (*p* < .001 for the difference between groups). The improvement in minutes of sleep with oxygen saturation < 90% from baseline to 6 months is displayed in Fig. 4 for each subject. Examination of hypoxemic burden as a percentage of sleep with oxygen saturation < 90% also demonstrated a significant improvement for treatment compared with control (Table 2). The percentage of sleep with oxygen saturation < 90% decreased by a median of 4 [− 10, 1] percentage points in the treatment group and the median in the control group changed by 0 [− 5, 5] percentage points at 6 months (between group *p* = 0.003).

More evidence of improved oxygen saturation during sleep included average oxygen saturation and lowest oxygen saturation. Average oxygen saturation throughout the night had a statistically significant increase in the treatment group compared with control (*p* = 0.006), with the treatment group average oxygen saturation improving by a median of 0.7 [− 0.4, 1.7] percentage points compared with control improving 0.1 [− 0.8, 0.8] percentage points at 6 months (Table 2). In addition to the treatment group experiencing fewer apnea and hypopnea events, the lowest oxygen saturation increased by a median of 2.0 [− 3.0, 5.0] percentage points from baseline for treatment and worsened 1.0 [−4.0, 3.0] percentage point for control at 6 months (*p* = 0.011) (Table 2).

The analyses were repeated in the HFrEF subgroup (26 treatment and 34 control subjects), yielding similar results as the full population for the sleep indices and nocturnal hypoxemia–related endpoints (Table 3). The change from baseline in minutes with oxygen saturation < 90% was statistically significant between treatment and control (median changes of − 21 [− 44, −4] min for treatment and 0 [− 31, 28] min for control, *p* = 0.037). The treatment group improved from a baseline median of 31 [16, 79] min to a median of 8 [1, 33] min at 6 months, whereas the control median was 27 [4, 65] min at baseline and increased to 34 [11, 65] minutes (Fig. 3B). The average oxygen saturation and lowest oxygen desaturation during the night also showed similar changes to those observed for the full population (Table 3).

**Table 3:** Median results for sleep parameters for overnight polysomnogram results by visit in the per protocol population with reduced ejection fraction heart failure

	Treatment (*n*=26)			Control (*n*=34)			Between group *P*-value
Variable	Baseline	6 Months	Change	Baseline	6 Months	Change	
AHI (events/hour)	47 41, 58	17 9, 35	−24 −38, −13	39 32, 52	41 33, 52	1 −4, 15	<.001
CAI (events/hour)	29 14, 41	1 0, 3	−22 −40, −14	20 14, 31	22 12, 30	−3 −9, 5	<.001
ODI4% (events/hour)	42 33, 53	17 7, 36	−21 −30, −7	34 26, 48	40 28, 51	3 −6, 14	<.001
Minutes of Sleep with O2	31	8	−21	27	34	−0	0.037
Saturation<90% (minutes)	16, 79	1, 33	−44, −4	4, 65	11, 65	−31, 28	
Percent of Sleep with O2	9	4	−6	9	12	−0	0.083
Saturation<90% (%)	5, 22	0, 17	−9, −2	2, 18	3, 26	−11, 8	
Average O2 Saturation	93.1	93.8	1.1	92.7	92.7	0.2	0.026
during sleep (%)	91.3, 94.4	92.3, 95.1	−0.4, 1.7	91.8, 94.5	91.5, 93.4	−1.6, 0.9	
Lowest O2 Saturation	81.0	81.5	1.0	82.0	80.0	−1.0	0.079
during sleep (%)	76.0, 83.0	76.0, 87.0	−3.0, 6.0	78.0, 85.0	75.0, 84.0	−6.0, 2.0	

Reported as median and interquartile range

*P*-value from Mann-Whitney test for difference in change from baseline between groups (2-sided)

*AHI* apnea-hypopnea index; *CAI* central apnea index; *ODI4* oxygen desaturation ≥4% index; *O*2 oxygen

## Discussion

This analysis is the first to show the improvement in T90 in patients with CSA treated with TPNS. This finding is particularly important as it has been previously shown that 22 min of oxygen saturation below 90% is associated with increased mortality in patients with heart failure and reduced ejection fraction and sleep apnea. In the current analysis, both the total population as well as the HFrEF population showed improvement of the median to below 22 min (Fig. 3) [3].

With the increased attention given to alternate endpoints for patients with CSA, namely, nocturnal oxygenation, that is clinically and prognostically more meaningful than AHI, it is promising to have therapeutic options in CSA able to improve measures of hypoxic burden such as delivered by TPNS therapy. Since TPNS activates automatically during sleep without need of patient intervention, TPNS delivers therapy throughout the night potentially providing more therapeutic treatment time than masked-based therapies and therefore potentially delivering more oxygenation improvement than other available therapies [9]. This is due to the fact that mask-based therapies require patient compliance and thus may not be used throughout the entire night, as is often the case in patients who have tolerability issues with wearing a mask-based therapy [15]. Additionally, the mask-based therapy ASV is contradicted in patients with HFrEF and CSA due to increased mortality in treated patients as shown in the SERVE-HF trial, leaving this subgroup with limited therapeutic options [7, 15].

Central sleep apnea has significant downstream effects including hypoxemia, hypocapnia and increased sympathetic tone along with chronic inflammation, endothelial dysfunction, and apoptosis [3, 16, 17]. Moreover, the atherosclerotic process, vascular and cardiac remodeling, and cardiac arrhythmias may be critical outcomes of these processes [16, 17]. While the improvement in AHI is associated with improvements in hypoxemia, time with oxygen saturation below 90% better characterizes the hypoxic burden and the possible downstream effects of intermittent hypoxia [3]. For example, the AHI is a frequency measure and thus does not account for the duration or depth of each apnea/hypopnea episode and does not differentiate patients with short episodes from those with the same number of longer episodes [3]. Further, a recent analysis demonstrated that a single metric of hypoxic burden, the oxygen desaturation “area under the curve,” that incorporates the frequency, duration, and depth of each respiratory event predicted cardiovascular disease mortality in patients with OSA [17]. However, custom software is necessary to calculate the area under the curve and therefore not commercially measured on polysomnograms at this point in time [18]. Thus, time below 90% is an important metric of disease burden in patients treated for sleep apnea, either OSA or CSA [3].

By design, the TPNS system allows some events (apneas/hypopneas) to persist in order to improve patient comfort and compliance [11]. For example, if a patient rolls over or takes a bathroom break, therapy will suspend and allow a window for the patient to return to sleep prior to enabling and ramping up to the therapeutic levels again. During the ramp period, patients may experience hypopneas rather than central apneas or events may be shorter in depth or duration as a result of the partially effective therapy delivered during this short period [11]. As shown in the **rem**edē System pivotal trial, TPNS significantly reduces central events (Fig. 2) and any apneas that persist are predominantly obstructive [12, 13]. The resulting events are associated with less hypoxia.

Oldenburg et al. demonstrated that T90 is associated with increased mortality in the HFrEF population with moderate-to-severe sleep-disordered breathing [3]. Based on this finding, TPNS may prove to be a critical tool in treatment of patients with CSA to prevent from detrimental hypoxia. The Oldenburg study showed that HFrEF patients with < 22 min of oxygen saturation below 90% had a lower risk of death than those with T90 higher than 22 min [3], and a recent community based study by Baumert supported this hypoxemia risk showing even T90 > 12 min in men indicated an elevated risk of CV mortality [19]. The **rem**edē System Pivotal Trial per protocol population showed the median T90 in the treatment group improved from 33 min at baseline to 14 min following 6 months of therapy [11]. In other words, the median minutes in the treatment group moved from above 22 min (a duration Oldenburg, et al. associated with worse outcomes) at baseline to <22 min at 6 months, while the control group showed no improvement (Fig. 3A) [11]. In the subgroup with HFrEF, the results were similar with median T90 in the treatment group improving from 31 min at baseline to 8 min following 6 months of therapy (Fig. 3B) [14]. Fifty percent of the Treatment group subjects who started with > 22 min of oxygen saturation improved to < 22 min at 6 months compared with 32% in the control group. Importantly, the median T90 went from above 22 min at baseline to below 22 min at 6 months and T90 even increased in the control group in both the full per protocol and HFrEF populations [14]. This is of particular interest because in the SERVE-HF trial the ASV treatment group suffered from a T90 median of 25 min after 48 months of treatment following no mortality benefit in the ASV treatment group [20].

Currently, there is an expansive scientific discussion ongoing whether Hunter–Cheyne Stokes respiration in heart failure is a friend or foe [21]. The so-called Naughton hypothesis postulates that Hunter–Cheyne Stokes respiration may be a potentially beneficial compensatory mechanism because hyperventilation increases end-expiratory lung volume and positive pressure augments cardiac stroke volume. Moreover, the hypothesis builds on attenuation of excessive sympathetic nervous activity and maintaining a state of respiratory alkalosis as a cardioprotective element. Finally, the provision of periodic rest relates to fatigue-prone respiratory pump muscles [21]. Nevertheless, various investigations do not support this hypothesis and recent literature on APAP and OSA showed that neither sleep quality nor sympatho-vagal balance is improved in HFrEF patients with OSA following APAP therapy [15, 22]. The lack of favorably altering the sympatho-vagal balance in HFrEF patients with CSA has also been shown for adaptive servoventilation therapy, which does not favorably alter sympatho-vagal balance in HFrEF patients with CSA either [23], which is further supported by a very recent publication by Spiesshofer et al., demonstrating that simulated CSR in heart failure patients does not decrease sympathetic drive [24]. For our study we conclude that even if the Naughton hypothesis was correct, nocturnal hypoxemia—which by the way is not entirely related to CSA only—is a promising therapeutic target besides the respiratory events of CSA. Our findings illustrate that relief from nocturnal hypoxemia through phrenic nerve stimulation may disburden these patients from the detrimental consequences of hypoxia.

Beyond guideline-derived recommendations, novel phrenic nerve stimulation may implicate a future perspective of sympathetic nerve affectation, which may include invasive microneurography measurements. For this approach TPNS may make a difference in addressing the pathophysiology of CSA, including hypoxia, more profoundly than mask-based therapy have the ability to. In this context it has been shown that positive airway pressure therapies have adversely impacted blood pressure and sympathetic nerve activity in heart failure patients [25]. Further investigations on phrenic nerve stimulation and effects on the sympatho-vagal system are needed using direct microneurography to assess physiologic interactions.

While the improvement in the lowest oxygen saturation overnight represents a single point in the night, the demonstrated improvements in average nocturnal oxygen saturation throughout the entire night and T90 support the idea that beneficial effects on cardiovascular outcome endpoints may be possible with this therapy in future trials.

This post hoc analysis has some limitations that need to be addressed. The current analysis includes a small sample size comprised primarily of white males. The sponsor acknowledges the gender and racial disparity and has initiated a large post market study (ClinicalTrials.gov Identifier: NCT03884660) and is committed to enrolling patients from subgroups that were underrepresented in the pivotal trial, including females, so more can be learned about the safety and effectiveness in underrepresented subgroups. Also, this analysis is constrained by not having long term follow-up data in the control group (control subjects had their TPNS device turned on after the 6-month assessment) that would allow analysis of mortality or of the impact of reducing T90 on survival between TPNS and control, as the trial was not powered to detect differences in mortality [11]. Future studies could consider assessing if reducing T90 with TPNS is tied to improved cardiovascular outcomes.

In conclusion, the use of nocturnal hypoxemic burden and measures related to desaturations are increasingly used by clinicians and are believed to have more clinical relevance than AHI and its sub-components as they do not indicate how significant an individual event is in each patient. While it is well known that apnea and hypopnea events have negative downstream effects, oxygenation may be more directly related to physiologic processes and therefore may represent a more comprehensible utility to clinicians. Reducing hypoxemic burden is a logical goal of any therapy that could possibly result in reduced mortality risk and transvenous phrenic nerve stimulation successfully improved oxygenation in the majority of patients in this trial, making it a promising option for CSA treatment, especially in subjects with HFrEF who are contraindicated for ASV therapy.
